# Blood glucose promotes dengue virus infection in the mosquito *Aedes aegypti*

**DOI:** 10.1186/s13071-021-04877-1

**Published:** 2021-07-26

**Authors:** Shih-Che Weng, Po-Nien Tsao, Shin-Hong Shiao

**Affiliations:** 1 Department of Tropical Medicine and Parasitology, College of Medicine, National Taiwan University, Taipei, Taiwan; 2 Department of Pediatrics, National Taiwan University Hospital, Taipei, Taiwan; 3 Research Center for Developmental Biology and Regenerative Medicine, National Taiwan University, Taipei, Taiwan

**Keywords:** *Aedes aegypti*, Dengue virus, Glucose, Signaling pathway

## Abstract

**Background:**

Dengue fever is the most rapidly spreading mosquito-borne viral disease globally. More than 2.5 billion people live in dengue-endemic areas. Previous studies suggested an interrelationship between diabetes mellitus (DM) and dengue hemorrhagic fever (DHF). Conversely, glycolysis is a critical metabolic pathway for optimal dengue virus (DENV) replication. However, little is known concerning the effect of glucose on DENV replication in mosquitoes. In this study, we investigated the impact of glucose on DENV replication in mosquitoes *Aedes aegypti*.

**Methods:**

Mosquitoes (*Ae. aegypti* UGAL/Rockefeller strain) were orally infected with DENV (serotype 2, 16681 strain) through infectious blood feeding. The DENV infection and transmission rates were determined by examining mosquito bodies and saliva, respectively, for DENV positivity at different time points after infection. In addition, a reverse genetic approach was applied by introducing double-stranded RNA against genes of interest into the mosquitoes to inhibit gene expression.

**Results:**

Our data revealed a significant increase of DENV genome levels in mosquitoes consuming an infectious blood meal supplemented with glucose, suggesting that blood glucose is an important factor for viral replication. Interestingly, a significant increase of DENV E protein levels was detected in the saliva 4 days faster in mosquitoes that consumed infectious blood meals supplemented with glucose than in those consuming infectious blood meals alone. Furthermore, we perform RNAi to silence AKT or TOR and investigate the molecular mechanism regulating the glucose-mediated enhancement of viral replication. Silencing of AKT or TOR significantly reduced DENV titers in mosquitoes.

**Conclusions:**

This study suggested that blood glucose is beneficial to DENV replication and that it facilitates virus transmission in mosquitoes via AKT and TOR signaling. Therefore, our results strengthen our understanding of dengue fever and DM co-morbidity and possibly reveal new targets for specific antiviral therapies.

**Graphical abstract:**

**Figure:**
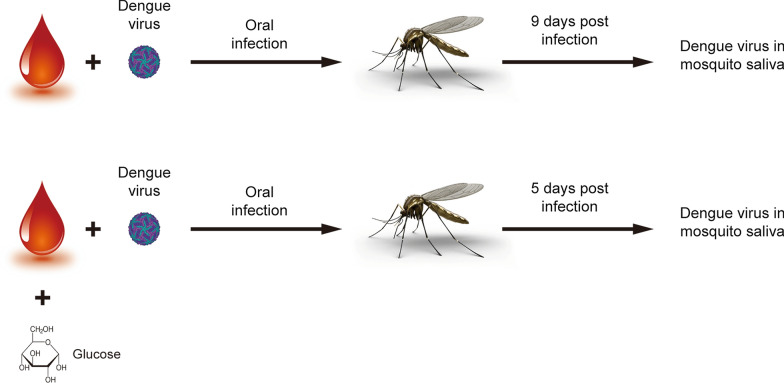

## Background

Several important and widespread infectious diseases, such as malaria, dengue fever, and Zika, are transmitted primarily by mosquitoes. Altogether, these diseases kill more than 1 million people annually, and more than 2 billion people are at risk worldwide [1]. Major reasons for this tragic situation are the unavailability of effective vaccines and drugs for most mosquito-borne diseases, increased resistance to insecticides in mosquitoes, and resistance of pathogens to currently available drugs [2].

Dengue fever is one of the most common arthropod-borne viral diseases globally, and it is caused by four dengue virus (DENV) serotypes (DENV1–4). DENV is a positive-stranded RNA virus belonging to the *Flaviviridae* family that is transmitted to humans primarily through the bite of infected *Aedes* mosquitoes. When the infected mosquitoes take a blood meal, they inject the infectious virus particle-containing saliva into human skin. The essential components of infectious viral particles are viral genome and viral structural proteins (envelope protein [E], membrane protein [M], and capsid protein [C]). A current estimate suggests that more than 390 million DENV infections occur annually [1, 2]. DENV infection causes several disease manifestations, ranging from undifferentiated fever and dengue fever to life-threatening dengue hemorrhagic fever/dengue shock syndrome (DHF/DSS) [1, 3, 4].

Diabetes mellitus (DM) is an abnormal endocrine disorder associated with abnormal glucose metabolism. Patients with DM generally have abnormally high blood glucose levels, resulting in several complications of hyperglycemia [3, 5–8]. In dengue-endemic areas, the prevalence of DM is also high, and previous studies revealed that dengue and DM can co-occur [6–11]. Interestingly, a few cell-based studies illustrated that during DENV infection, the glycolytic pathway of glucose metabolism is induced to promote efficient viral replication [12–14]. A retrospective cohort study of adults with DM and acute dengue infection indicated that the oral antidiabetic drug metformin can attenuate disease severity in individuals with co-morbid dengue infection and DM [8]. These findings indicated that glucose metabolism may be important for dengue infection, DHF, and mortality.

Glucose is the most abundant monosaccharide in humans, and it plays key roles in cell growth and metabolic regulation. Many viruses modulate signaling pathways to regulate virus replication. AKT and TOR signaling pathways were shown to be important in various cellular processes, such as metabolic regulation and cell growth, proliferation, and survival [15, 16]. These pathways are also highly conserved among different species ranging from an early branching eukaryote to mammals [15, 16]. Previous studies indicated that during viral infection, cells initiate the stress response to limit viral spread. The activation of TOR inhibits both apoptosis and stress-induced autophagy. Therefore, viruses have evolved to maintain a basal level of activity along the PI3K/AKT/TOR pathway [15–18]. Viruses in the genus *Flavivirus*, such as DENV, Zika virus (ZIKV), West Nile virus (WNV), and Japanese encephalitis virus (JEV), hijack the PI3K/AKT/TOR pathway to promote their successful replication in mammalian cells [19–24]. In mosquitoes, the PI3K/AKT/TOR pathway promotes Sindbis virus infection [25]. The replication complex formation induces PI3K/AKT/TOR pathway upregulation and 4E-BP1 phosphorylation, promoting cap-dependent translation in mosquito cells [25]. AKT and TOR are also involved in mosquito egg production, immune responses, and survivorship [25–30].

In this study, we investigated the possibility that blood glucose promotes DENV replication and facilitates viral transmission in mosquitoes via AKT and TOR signaling. Our data revealed a significant increase of DENV genome levels in mosquitoes consuming an infectious blood meal supplemented with glucose, suggesting the importance of blood glucose for viral replication. Interestingly, a significant increase of DENV E protein levels was detected in the saliva 4 days earlier in mosquitoes consuming infectious blood meals supplemented with glucose than in those consuming infectious blood meals alone. Furthermore, silencing of AKT or TOR significantly suppressed DENV titers in mosquitoes.

## Methods

### Mosquitoes

Mosquitoes (*Ae. aegypti* UGAL/Rockefeller strain) were kept at 28 °C and 70% relative humidity under a 12 h:12 h light–dark cycle as previously described [31]. Hatched larvae were transferred to plastic containers with sufficient water and fed yeast extract daily. Pupae were collected and transferred to a plastic container in an insect dorm. Emerged mosquitoes were fed using cotton balls soaked in 10% sucrose solution. Female mosquitoes were used for our experiments 3–5 days post-eclosion (PE). The sucrose-soaked cotton balls were removed at least 12 h before blood-feeding. Female mosquitoes were permitted to blood-feed on an anesthetized ICR strain mouse for 15–30 min. ICR strain mice were anesthetized via an intraperitoneal injection of avertin at a dose of 0.2 ml/10 g. All animal procedures and experimental protocols were approved by an AAALAC-accredited facility and the Committee on the Ethics of Animal Experiments of the National Taiwan University College of Medicine (IACUC approval No: 20200210).

### Cell culture and virus

*Aedes albopictus* C6/36 and *Ae. aegypti* CCL-125 cells were cultured in Dulbecco's Modified Eagle's Medium (DMEM) (calcium chloride dihydrate 265 mg/l, ferric nitrate nonahydrate 0.1 mg/l, magnesium sulphate anhydrous 97.720 mg/l, potassium chloride 400 mg/l, sodium chloride 6400 mg/l, glycine 30 mg/l, l-arginine hydrochloride 84 mg/l, l-cystine dihydrochloride 62.57 mg/l, l-glutamine 584 mg/l, l-histidine hydrochloride monohydrate 42 mg/l, l-isoleucine 105 mg/l, l-leucine 105 mg/l, l-lysine hydrochloride 146 mg/l, l-methionine 30 mg/l, l-phenylalanine 66 mg/l, l-serine 42 mg/l, l-threonine 95 mg/l, l-tryptophan 16 mg/l, l-tyrosine disodium salt 103.79 mg/l, l-valine 94 mg/l, choline chloride 4 mg/l, d-calcium pantothenate 4 mg/l, folic acid 4 mg/l, nicotinamide 4 mg/l, pyridoxal hydrochloride 4 mg/l, riboflavin 0.4 mg/l, thiamine hydrochloride 4 mg/l, i-inositol 7.2 mg/l, D-glucose 4500 mg/l, phenol red sodium salt 15.9 mg/l) and Mitsuhashi and Maramorosch Insect Medium (MM) (calcium chloride dihydrate 190 mg/l, magnesium chloride anhydrous 46.9 mg/l, potassium chloride 200 mg/l, sodium chloride 7000 mg/l, sodium phosphate monobasic 173.9 mg/l, D( +) glucose 4000 mg/l, lactalbumin hydrolysate 6500 mg/l, yeast extract 5000 mg/l) in a 1:1 ratio containing 2% heat-inactivated fetal bovine serum and 1% penicillin–streptomycin solution. For virus production, C6/36 cells were infected with the DENV2 strain 16681 at a multiplicity of infection of 0.01. The culture supernatant was harvested on day 7 post-infection and stored at −80 °C. To determine the viral titer, the virus stock was subjected to examination using a plaque assay, as previously described [31]. Approximately 1.0 × 10^7^ PFUs/ml DENV2 were used to infect the mosquitoes. To examine the role of ATK and TOR signaling pathways in linking glucose and DENV replication in *Ae. aegypti* CCL-125 cells, cell culture medium containing different glucose levels were prepared.

### Oral infection of mosquitoes and mosquito saliva collection

Infection of mosquitoes was achieved through an infectious blood meal via folded Parafilm-M. After starvation through sugar deprivation for 24 h, female mosquitoes were subsequently provided an infectious blood meal prepared by mixing 200 μl of mouse whole blood, 50 μl of 1 mM ATP, and 250 μl of DENV2 16,681 (2.5 × 10^6^ PFU in 250 μl). After the blood feeding, each mosquito was examined on a stereo microscope to determine whether it had taken a full meal. Mosquitoes kept at 28 °C and 70% relative humidity under a 12 h:12 h light–dark cycle as previously described [31, 32].

To collect saliva, female mosquitoes were starved for 24 h prior to saliva collection. On the day of saliva collection, the feeding solution (ATP-containing phosphate-buffered saline (PBS)) was wrapped in stretched Parafilm-M membrane and put on the top of a container covered with nylon mesh, allowing mosquitoes to feed on the meal. The mosquito saliva-containing solution was removed from the membrane and transferred to a microtube and centrifuged at 12,000×*g* for 1 min at 4 °C. The protein concentration of mosquito saliva was measured using Bradford protein assays [31, 32].

### RNA extraction and reverse transcription (RT)

The whole bodies of 3–5 mosquitoes were collected in 1.5-ml tubes containing 0.5 ml of TRIzol (Invitrogen). Tissue was homogenized using a rotor–stator homogenizer at room temperature for 5 min and centrifuged at 15,890×*g* or 10 min at 4 °C. After centrifugation, the supernatant was transferred to a new micro-tube containing 0.1 ml of chloroform (J. T. Baker) and mixed thoroughly at room temperature for 3 min. Samples were then centrifuged at 15,890×*g* or 15 min at 4 °C, and the supernatant was transferred carefully to a new micro-tube containing 0.25 ml of isopropanol (J. T. Baker). Samples were gently mixed and stored at −80 °C for 30 min. After precipitation, the samples were again centrifuged at 15,890×*g* or 30 min at 4 °C. The supernatant was discarded, and 0.5 ml of 75% ethanol (Taiwan Burnett International Co., Ltd) was used to wash the RNA pellet. All resulting samples were centrifuged at 15,890×*g* or 5 min at 4 °C, and the supernatant was discarded. Finally, the RNA pellet was dried in a laminar flow hood and dissolved in DEPC-H_2_O. After Baseline-ZEROTM DNase (Epicenter) treatment, the RNA sample was stored at −80 °C.

The RNA concentration was quantified using a spectrophotometer (Nanodrop 2000, Thermo Fisher Scientific), and the sample was diluted with DEPC-H_2_O to a concentration of 1 μg/μl. The RNA samples were reverse-transcribed to cDNA using a High-Capacity cDNA Reverse Transcription Kit (Applied Biosystems). The cDNA samples were stored at −20 °C for further use. Gene expression was analyzed via quantitative polymerase chain reaction (qPCR). The ribosomal protein S7 gene was used as an internal control.

### Quantitative PCR (qPCR)

The SYBR Green dye binding system was used for qPCR in this study. SYBR Green binds the minor groove of DNA, and the target gene expression was quantified by detecting the resulting fluorescence signal. The cDNA sample was quantified using a KAPA SYBR FAST Universal qPCR kit (KAPA), via the qPCR primers (S7: 5′-TCAGTGTACAAGAAGCTGACCGGA-3′/5′-TTCCGCGCGCGCTCACTTATTAGATT-3′; DENV:5′-GAAGACATTGACTGYTGGTGCAA-3′/ 5′-CGATGTTTCCACGCCCCTTC-3′). The PCR protocol consisted of initial denaturation at 95 °C for 3 min, followed by 40 cycles of 3 s at 94 °C and 40 s at 60 °C. Fluorescence readings were measured at 72 °C after each cycle. The target gene signal was detected and analyzed using the ABI 7900HT Fast Real-Time PCR System, and relative quantification results were normalized using the ribosomal protein S7 gene as an internal control.

### Double-stranded RNA (dsRNA) preparation

RNAi primers were designed using the E-RNAi webservice (http://www.dkfz.de/signaling/e-rnai3//). The T7 promoter sequence (5′-TAATACGACTCACTATAGGG-3′) was incorporated into all forward and reverse RNAi primers (TOR RNAi: 5′-TAATACGACTCACTATAGGGCCAAGCGTGGGATTTGTACT-3′/ 5′-TAATACGACTCACTATAGGGAGATGGTCGTATCCGTTGC-3′; AKT RNAi: 5′-TAATACGACTCACTATAGGGACCAGATTTTATGGCGCAGA-3′/ 5′-TAATACGACTCACTATAGGGGTTAGCTCGACGCTTTCACC-3′). The target gene fragment was amplified using Ex Taq DNA Polymerase (Takara). Fragments were amplified and cloned into a pCR 2.1-TOPO vector at 23 °C for 30 min using a TOPO TA Cloning Kit (Invitrogen). The constructed plasmid was transformed into HIT-DH5α competent cells. Plasmids from positive colonies were purified using a FarvoPrep™ Plasmid DNA Extraction Mini Kit (Favogen) and sequenced to confirm that the cDNA was in frame.

The plasmid was digested by a restriction enzyme, and fragments were separated using 1% agarose gel. Target fragments were isolated and purified from the gel using a FarvoPrep™ GEL/PCR Purification Kit (Favogen). The fragments were then amplified using Ex Taq DNA Polymerase and purified using a FarvoPrep™ GEL/PCR Purification Kit. The purified PCR product was used as the template to synthesize dsRNA in vitro using a T7-Scribe™ Transcription Kit (Epicenter). The reaction was performed at 37 °C for 4–12 h. A solution of 95 μl of DEPC-H_2_O and ammonium acetate (stop solution) was added to stop the reaction, and the supernatant was transferred into a new Eppendorf tube containing 150 μl of a phenol/chloroform (Amresco) solution. Samples were centrifuged at 15,890×*g* or 5 min, at 4 °C, and the supernatant was transferred to a new Eppendorf tube containing 150 μl of chloroform. After centrifugation at 15,890×*g* or 5 min at 4 °C, the supernatant was transferred to a new Eppendorf tube containing 110 μl of isopropanol. Samples were gently mixed and stored at −80 °C for 30 min. Finally, each sample was centrifuged at 15,890×*g* or 30 min at 4 °C. The dsRNA pellets were dried in a laminar flow hood and dissolved in DEPC-H_2_O.

The dsRNA was diluted to a final concentration of 5 μg/μl. Between day 3 and 5 PE, female mosquitoes were injected with 280 nl of dsRNA (5 μg/μl) using a Nanoject II AutoNanoliter Injecter (Drummond Scientific Company). dsRNA against LacZ was used as the control dsRNA (dsLacZ). Silencing efficiency was confirmed by collecting the total RNA of mosquitoes 3 days post-injection for RT-PCR.

### Western blot analysis

CCL-125 cells were collected in 1.5-ml Eppendorf tubes containing 100 µl of protein lysis buffer and homogenized using a rotor–stator homogenizer. Each homogenized sample was centrifuged at 15,890×*g* or 30 min at 4 °C, and the supernatant was transferred to a QIAshredder™ column (Qiagen). The eluted samples were collected and transferred to new Eppendorf tubes at −80 °C. The protein concentration was quantified using the Bradford method and Bio-Rad Protein Assay Dye Reagent (Bio-Rad Laboratories, Inc). Each protein sample was mixed with the same volume of sample buffer Laemmli 2× Concentrate (Sigma-Aldrich) and adjusted to the same volume with 1× sample buffer. To denature proteins for electrophoresis, protein samples were incubated at 98 °C for 18 min. The protein samples (10 µg) were subjected to SDS-PAGE and blotted onto a PVDF membrane (Pall Corporation) for 1.5 h. The membranes were blocked with 5% skim milk in 1× phosphate-buffered saline containing 0.4% Tween 20 (PBST) at room temperature for 1 h. Afterward, the membranes were incubated in PBST containing primary antibody overnight at 4 °C. The antibodies used in this study were: mouse anti-NS1 (Yao-Hong Biotechnology, YH0023, 1/10000), rabbit anti-NS5 (GeneTex, GTX103350, 1/1000), mouse anti-E (Yao-Hong Biotechnology, YH0026, 1/10,000), mouse anti-prM (1/100), rabbit anti-pS6K (Merck, 07-018, 1/1000), rabbit anti-S6K (Santa Cruz Biotechnology, sc-230, 1/1000), rabbit anti-pAKT (Cell Signaling, 9271, 1/1000), rabbit anti-AKT (Cell Signaling, 9272, 1/1000), and rabbit anti-GAPDH (GeneTex, GTX100118, 1/10,000). Membranes were washed in PBST and incubated with secondary antibody (HRP-conjugated anti-mouse IgG, or HRP-conjugated anti-rabbit IgG) in PBST at room temperature for 1 h. Finally, membranes were washed in PBST and developed using WesternBright™ Peroxide and ECL (Advansta Inc.) as the substrate for horseradish peroxidase following the manufacturer’s instructions.

### Plaque assay

The whole bodies and saliva of TOR-silenced, AKT-silenced, or dsLacZ-treated mosquitoes were collected in 100 μl of serum-free medium and stored at −80 °C. C6/36 cells were seeded in a 24-well tissue culture plate and incubated at 28 °C overnight. The homogenized suspensions of infectious mosquitoes were centrifuged at 18,928×*g* for 30 min and kept on ice. The cell monolayers were rinsed with PBS, and 200 μl of the tenfold serial dilutions of infectious mosquito suspensions were added for 2 h. After viral adsorption, 500 μl of 1% methylcellulose cell medium were added, and the plates were kept in an incubator at 28 °C for 5 days. The plates were fixed with 4% formaldehyde for 1 h at room temperature and stained with 1% crystal violet for 30 min. Plaques were quantified via manual counting as described previously [31].

### Statistical analysis

All statistical analyses were performed using GraphPad Prism 5 software. Gene expression and fecundity data were analyzed using ANOVA for all independent experiments.

## Results

### The glucose concentration is an important factor for DENV

**Fig. 1:**
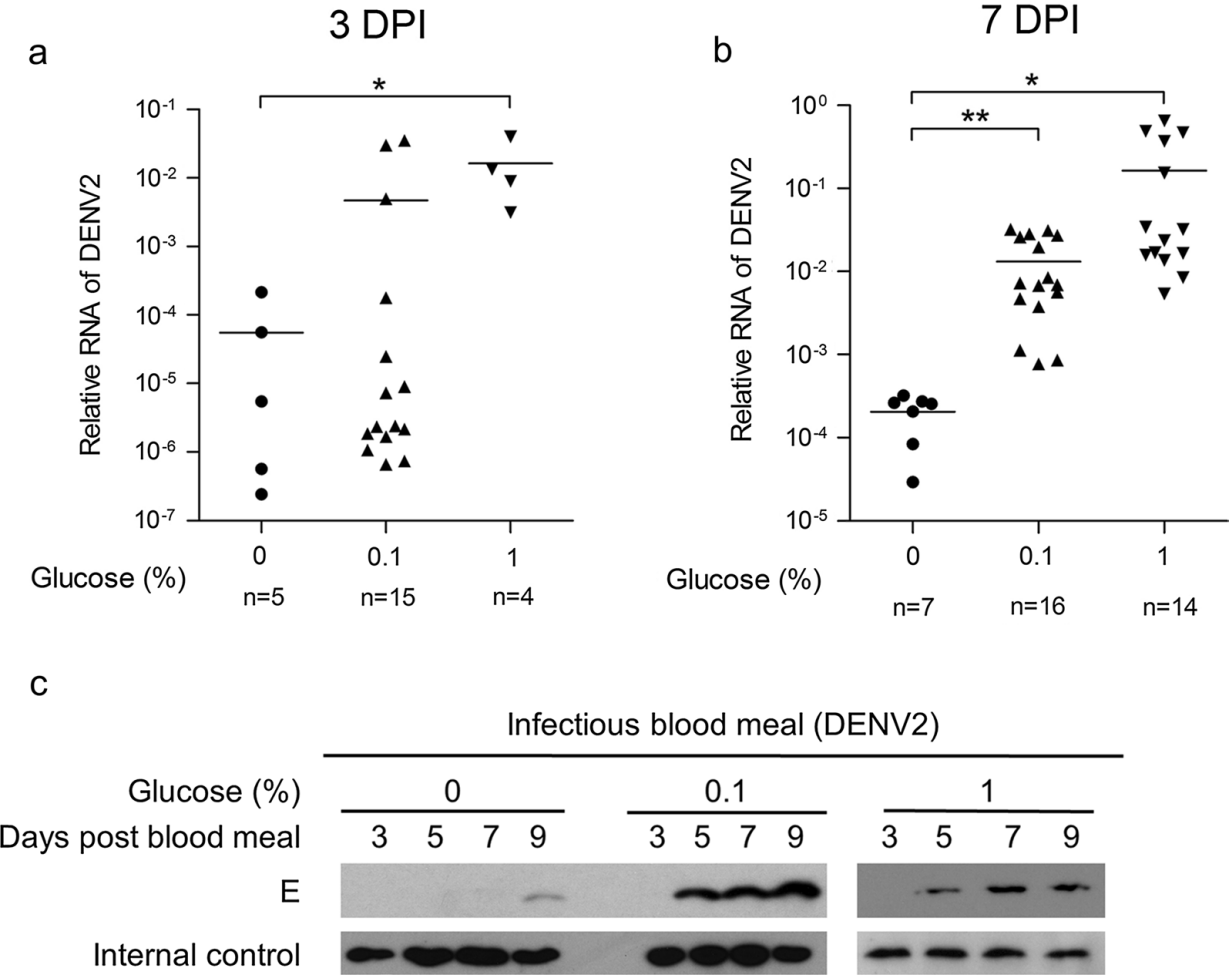
Blood meals supplemented with glucose are beneficial for DENV replication and transmission in *Aedes aegypti*. The total RNA of individual whole mosquitoes was collected 3 (**a**) and 7 (**b**) days after blood meal consumption. The relative levels of the DENV genome were quantified using real-time PCR analysis. Numbers denotes total number of infected mosquitoes. All examined groups were demonstrated to be statistically significant using ANOVA; *P < 0.05, **P < 0.01. **c** Salivary proteins were collected from pools of hundreds of mosquitoes at 3, 5, 7, and 9 days after the consumption of infectious blood meals with or without additional supplementation of 0.1% and 1% glucose. E protein expression was determined via western blotting. Purified serum from mouse routinely bitten by naïve mosquitos was used to serve as a primary antibody for internal control of total salivary proteins

We first examined the copy number of viral genomes in mosquitoes after taking infectious blood meals containing additional glucose. Our data indicated that the copy number of viral genomes was increased at 3 and 7 days post an infectious blood meal containing high glucose (Fig. 1a, b). Interestingly, the production of dengue virus E protein in the mosquito saliva was revealed 4 days earlier and higher when mosquito consumed a blood meal containing additional glucose than without additional supplementation of glucose (Fig. 1c). Taken together, our results indicated that both replication of the viral genome and viral E protein production were increased when mosquitoes consumed a blood meal containing an elevated glucose level.

### AKT and TOR signaling are activated during mosquito cell incubation in high glucose medium

**Fig. 2:**
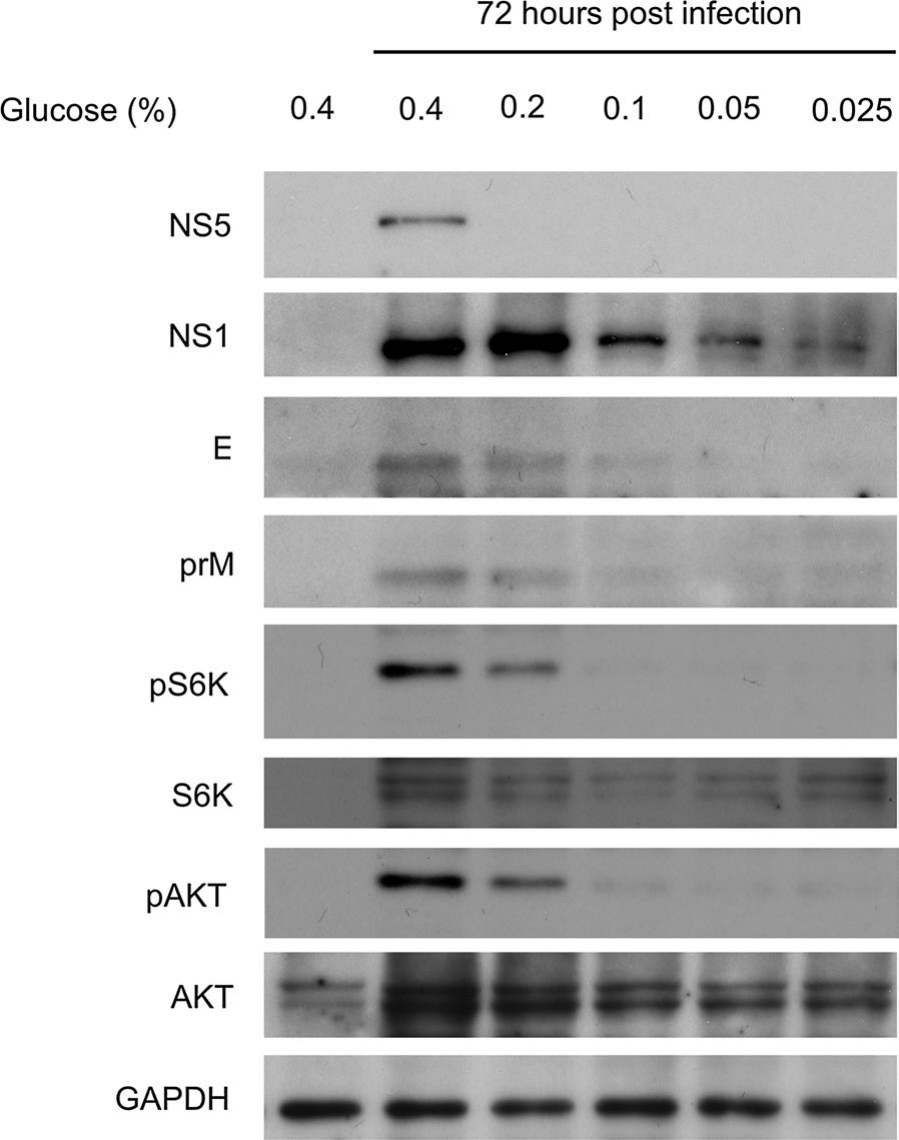
AKT and TOR pathways are activated during DENV infection in a glucose-dependent manner. *Aedes aegypti* cells (CCL-125) were infected with DENV at a multiplicity of infection (MOI) of 1 with different concentrations of glucose. Total protein was collected 72 h after infection. TOR and AKT pathway activation and DENV protein (prM, E, NS1, and NS5) expression were determined via western blotting. Human GAPDH served as a loading control. Three biological cohorts were analyzed

Our data showed that the DENV proteins NS1, NS5, E, and prM, AKT signaling (AKT phosphorylation), and TOR (S6K phosphorylation) signaling were upregulated in mosquito cells incubated in high glucose medium (Fig. 2). These findings suggested the involvement of AKT and TOR signaling in DENV replication in a glucose concentration-dependent manner.

### AKT and TOR signaling are involved in the glucose-dependent enhancement of DENV replication and transmission in mosquitoes

**Fig. 3:**
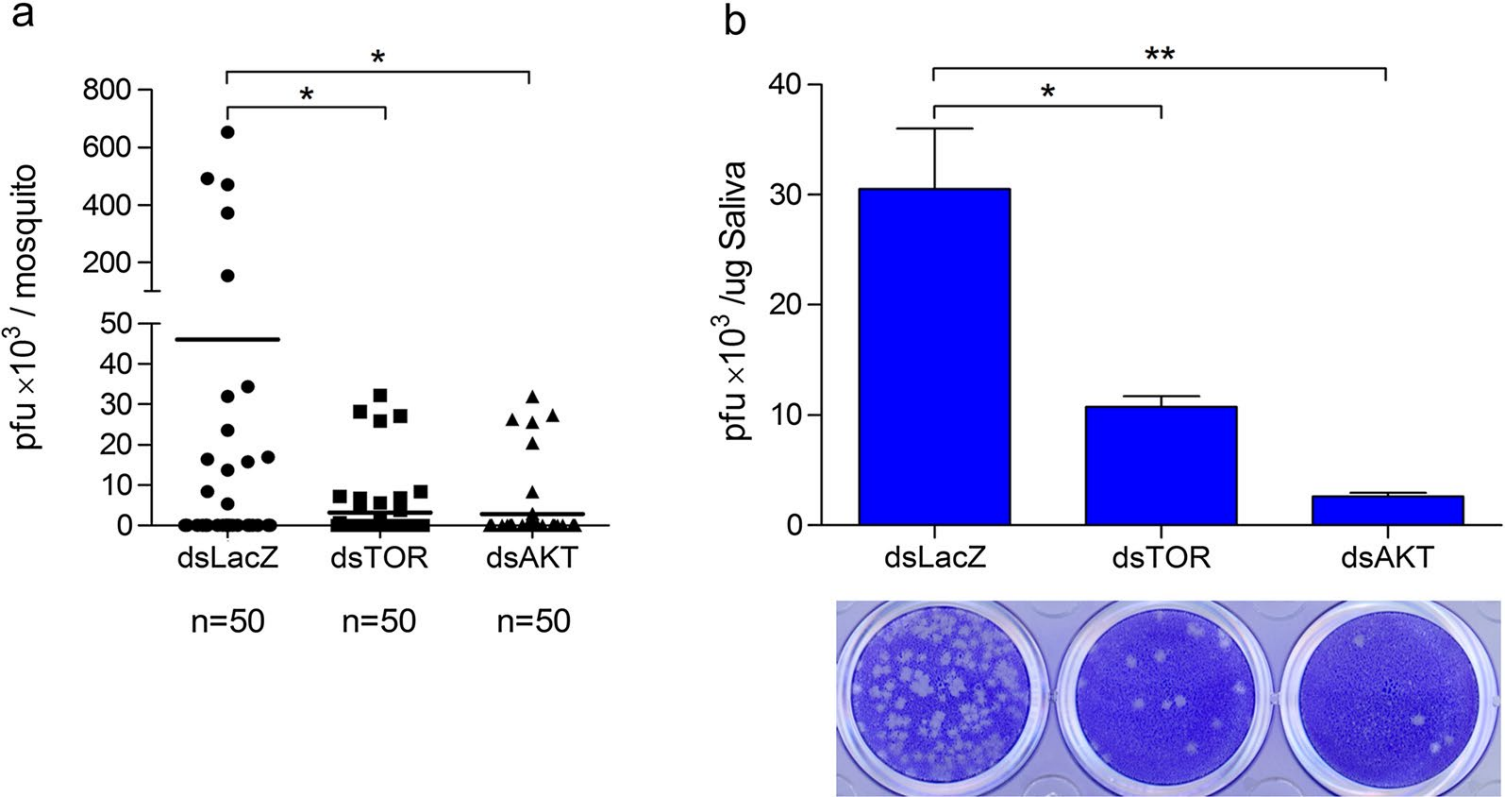
AKT and TOR pathways are essential for DENV replication and transmission in *Aedes aegypti.* Three-day-old female mosquitoes were injected with dsRNA for LacZ (dsLacZ), AaTOR (dsTOR), or AaAKT (dsAKT) 3 days prior to the consumption of DENV infectious blood meals (1 × 10^7^ pfu/ml) containing 0.1% glucose. **a** Nine days after infectious blood meal consumption, the viral titers of individual mosquitoes were quantified using a plaque assay. Numbers denotes total number of mosquitoes examined. **b** Nine days after infectious blood meal consumption, saliva was collected from pools of hundreds of mosquitoes and quantified using a plaque assay. All examined groups were demonstrated to be statistically significant using unpaired *t*-test; *P < 0.05, **P < 0.01. Data shown are from three independent biological cohorts and are presented as the mean ± SEM

Our data revealed that the DENV titers of individual mosquitoes and their saliva were decreased after the AKT or TOR gene was silenced (Fig. 3). These findings indicated the potential involvement of AKT and TOR signaling in DENV replication and transmission during the consumption of blood meals containing high blood levels by mosquitoes.

## Discussion

Previous studies found that circulating sugar levels change dramatically during blood-feeding by mosquitoes [33]. Some studies also revealed that dengue and diabetes mellitus can co-occur [3, 7, 34–39]. At the cellular level, glucose concentrations and metabolism contribute to DENV replication [12–14]. In this study, we observed that DENV replication and transmission rates are enhanced when mosquitoes consume an infectious blood meal with high glucose content, and these changes are dependent on AKT and TOR signaling.

The family *Flaviviridae* comprises several genera of viruses, including *Flavivirus*, *Hepacivirus*, *Pestivirus*, and *Pegivirus*. Hepatitis C virus (HCV) is a *Hepacivirus* species linked to insulin resistance and the subsequent development of type 2 diabetes [40, 41]. Previous studies indicated that in the United States, type 2 diabetes is more prevalent in HCV-infected people older than 40 years of age, and HCV infection accelerates the course of diabetes, with the disease developing a decade earlier than observed in uninfected people [42]. Meanwhile, some studies indicated that the HCV core protein is involved in the induction of insulin resistance through insulin receptor substrate-1 pathway signaling [40]. There is substantial overlap between dengue fever-endemic regions and regions with an increasing prevalence of diabetes [5, 7, 34, 37, 42, 43]. Moreover, according to linear regression analyses, approximately 10–11.4% of the variation of the prevalence of diabetes can be attributed to the dengue burden [42]. A small-scale study conducted in a highly DENV-endemic region indicated that 75% of patients developed glucose intolerance during early infection, and 17.5% of patients presented with persistent glucose intolerance post-infection [39].

At the cellular level, DENV infection increases the cellular capacity of glucose metabolism and plays an anaplerotic role in the oxidation of endogenous fatty acids, which are the main energetic substrate during infection [12]. Clinico-epidemiological data and results from type 2 diabetes mouse models also indicated that type 2 diabetes is associated with an increased risk of WNV encephalitis [44–47]. Furthermore, in a type 2 diabetes mouse model, lower expression of cell adhesion molecules reduced leukocytes recruitment, resulting in a failure to clear WNV infection from the brain and leading to increased inflammatory molecule levels, which increases neuronal cell death and mortality in mice with type 2 diabetes [48, 49]. Some studies also reported that the children of mothers who are diabetic (pre-pregnancy or gestational) are at higher risk for both explained and unexplained microcephaly during ZIKV infection [50, 51]. Taken together, several members of *Flaviviridae* including DENV appear to be associated with diabetes. Interestingly, mosquito-borne diseases such as WNV, yellow fever virus, and Zika are also associated with diabetes. Our data revealed that glucose is important for DENV replication and transmission in *Ae. aegypti* females. These data indicated that people with diabetes have a higher risk of life-threatening DHF/DSS and a greatly likelihood to transmit the virus to mosquito vectors.

Many viruses modulate signaling pathways to regulate viral replication. Previous studies illustrated that during viral infection, cells initiate the stress response to limit viral spread. The activation of TOR inhibits both apoptosis and stress-induced autophagy. Therefore, viruses have evolved to maintain a basal level of activity along the PI3K/AKT/TOR pathway [15, 16]. Members of *Flavivirus*, such as DENV, ZIKV, WNV, and JEV, have been demonstrated to hijack the PI3K/AKT/TOR pathway to promote their successful replication in mammalian cells [15, 18–21, 24]. Based on our result, we found that AKT and TOR signaling are involved in the glucose-dependent enhancement of DENV replication and transmission in mosquitoes.

In arthropods, AKT and TOR signaling also play an important role in viral replication [17, 25, 52]. During white spot syndrome virus infection in shrimp, the PI3K/AKT/TOR pathway is activated, and several metabolic pathways associated with the vertebrate Warburg effect, including glycolysis, the pentose phosphate pathway, nucleotide biosynthesis, glutaminolysis, and amino acid biosynthesis, are significantly upregulated to facilitate successful viral replication [53–55]. Flock house virus-infected flies have a dose-responsive loss of fecundity that corresponds to a global reduction of AKT/TOR signaling [25]. However, during Sindbis virus infection in flies, AKT signaling is activated, and it is important for viral replication [25].

In mosquitoes, the PI3K/AKT/TOR pathway promotes Sindbis virus infection [25]. The replication complex formation induces PI3K/AKT/TOR pathway upregulation and 4E-BP1 phosphorylation, promoting cap-dependent translation in mosquito cells [25]. AKT and TOR are also involved in mosquito egg production, immune responses, and survivorship [25–30]. However, the correlation between glucose and PI3K/AKT/TOR pathway activity during DENV infection remains to be further investigated.

## Conclusions

In this study, we investigated the possibility that blood glucose promotes DENV replication and facilitates viral transmission in mosquitoes via AKT and TOR signaling. Our results revealed a significant increase of DENV genome levels in mosquitoes consuming an infectious blood meal supplemented with glucose, suggesting the importance of blood glucose for viral replication. Interestingly, a significant increase of DENV E protein was detected in the saliva 4 days earlier in mosquitoes consuming infectious blood meals supplemented with glucose than in those consuming infectious blood meals alone. Furthermore, silencing of AKT or TOR significantly suppressed DENV titers in mosquitoes.

## Data Availability

All data generated or analysed during this study are included in this published article.

## Abbreviations

DENV: Dengue virus
DF: Dengue fever
DHF: Dengue hemorrhagic fever
DM: Diabetes mellitus
HCV: Hepatitis C virus
JEV: Japanese encephalitis virus
PE: Post-eclosion
WNV: West Nile virus
ZIKV: Zika virus
